# If at first you don’t succeed … adoption of iPad marking for high-stakes assessments

**DOI:** 10.1007/s40037-017-0372-y

**Published:** 2017-08-17

**Authors:** Terry Judd, Anna Ryan, Eleanor Flynn, Geoff McColl

**Affiliations:** Department of Medical Education, The University of Melbourne, Melbourne, Australia

**Keywords:** Mobile assessment, OSCE, MMI, Adoption of technology

## Abstract

Large-scale interview and simulation-based assessments such as objective structured clinical examinations (OSCEs) and multiple mini interviews (MMIs) are logistically complex to administer, generate large volumes of assessment data, and are strong candidates for the adoption of computer-based marking systems. Adoption of new technologies can be challenging, and technical failures, which are relatively commonplace, can delay and/or create resistance to ongoing implementation.

This paper reports on the adoption process of an electronic marking system for OSCEs and MMIs following an unsuccessful initial trial. It describes how, after the initial setback, a staged implementation, progressing from small to larger-scale assessments, single to multiple assessment types, and lower to higher stakes assessments, was used to successfully adopt and embed iPad-based marking within our medical school.

Critical factors in the success of this approach included thorough appraisal and selection of technologies, rigorous assurance of system reliability and security, constant review and refinement, and careful attention to implementation and end-user training. Engagement of stakeholders is also crucial, especially in the case of previous failures or setbacks. The early identification and recruitment of staff to provide specific expertise and support for adoption of an innovation helps to facilitate this process with four key roles proposed; those of innovation advocate, champion, expert and sponsor.

## Introduction

Interview and simulation-based assessments, which include objective structured clinical examinations (OSCEs) and multiple mini interviews (MMIs), are widely used in medical education settings but are administratively and logistically complex and expensive to deliver. OSCE and MMI marking is traditionally paper-based, employing structured assessment rubrics. Marking of these forms takes time, and is prone to security and reliability issues, including incomplete or missing forms and transcription errors. Electronic marking systems can dramatically reduce manual handling of assessments, minimizing errors and expediting the provision of results and feedback [1–3]. Studies also suggest that examiners may prefer the convenience of electronic marking [2] and provide richer feedback compared with paper forms [3]. There are a number of commercial OSCE marking tools, but to our knowledge no specialized MMI marking systems are available.

The case for adopting electronic marking for OSCEs and MMIs appears straightforward. However, adoption of new technologies is often complex and challenging, and for high-stakes assessments the consequences of system failure are severe. Common barriers to successful adoption include technical issues and administrative constraints. Technical success implies that the selected technology exhibits high levels of utility and usability and is compatible with existing systems. Administrative success is more typically concerned with human processes, such as personnel and expertise to implement the technology and change management. Institutional and personal attitudes to technology add an additional layer of complexity to the adoption process.

While there are a range of approaches to managing and understanding technology adoption, we argue that it is easiest to think of this in terms of three key processes:

1. Selection – establishing the need for a solution, gathering user requirements and selecting (and perhaps modifying) an existing tool, or developing a new one;
2. Implementation – introducing the technology into practice, and
3. Evaluation – assessing success and impact.

These processes are not exclusive and often overlap. A successful adoption design carefully considers all three processes, and is flexible enough to respond to changing circumstances or requirements. The relative focus on each of the three stages will also vary according to the type and scale of the adoption. Adoptions that involve the development of new technologies or substantial adaptations of existing technologies tend to be more complex and drawn out. Iteration is a common feature of many successful technology adoptions and can involve prototyping and pilot testing during selection, staged or incremental rollouts during implementation and regular and ongoing evaluation of utility, acceptance and impact.

### Adoption studies and theories

Rather than focusing on the entire adoption process, most studies involving technology adoption in education have tended to consider only one or a few elements. User acceptance is a common theme, and the Technology Acceptance Model and its variants are popular frameworks for analyzing user acceptance data, within a context of the target technology’s perceived usefulness and perceived ease-of-use [4, 5]. Technology uptake is another common theme. Best embodied by Rogers’ diffusion of innovations theory [6], it characterizes the adoption of technology based on the speed and scale of its uptake by end users. Often visualized as an ‘adoption-time curve’ it characterizes the earliest adopters who shape the initial portion of the curve as innovators with the tail of the curve representing the ‘laggards’. However, both the Technology Acceptance Model and diffusions of innovation theory focus on adoption by end users, and may be less relevant in cases of institutional (versus personal) adoption of educational technologies.

The distinction between personal and institutional adoption is an important one here because for electronic marking systems, most end users will have little or no say over when or how the system is utilized. This can lead to users, who Rogers would characterize as ‘laggards’, being forced into the role of innovators. Imposing technology on these users can lead to a degree of adoption resistance and anxiety. However, as previously indicated, adoption problems are much more likely to result through technical or organizational setbacks or failures [7]. If these setbacks are sufficient to substantially interrupt the selection or implementation of a technology, then acceptance can also suffer, and may even lead to active resistance to continued or future adoption [8]. Overcoming such resistance is challenging and necessitates careful planning and change management.

## The adoption of an electronic marking system: a case study

Our medical school’s adoption of electronic marking systems for OSCEs and MMIs showcases an adoption process designed to overcome a substantial setback. While our implementation of electronic marking is by no means unique, we believe that detailing our adoption process, through initial failure, subsequent resistance and gradual reintroduction and consolidation, is informative. And, in doing so we develop useful suggestions for medical educators and decision-makers contemplating technology adoptions that are similar either in context or scale.

## Stage 1: The setback (OSCE marking – unsuccessful)

A trial of electronic marking of OSCEs on iPads was conducted within our department during 2012, as part of a larger university-funded project. This trial involved a mock OSCE of six stations with 24 volunteer student participants and utilized a commercial browser-based OSCE marking tool. A post-trial evaluation found, consistent with another study involving this tool [2], that users were generally satisfied, found it easy to use and preferred it to paper-based marking. However, a serious technical issue, related to intermittent WiFi connectivity, resulted in some loss of functionality and data loss. The project was discontinued and no further trials of electronic marking for OSCEs or similar assessments were conducted within our department during the next three years.

The failure of this initial trial created resistance towards electronic marking. Reintroducing it would require a measured approach.

## Stage 2: The solution part 1 (MMI marking)

An opportunity to begin this process arose in 2014, during conversations between two of the authors. One of these – EF, who has responsibility for our MMI program – had recently observed a successful implementation of iPads-based marking, and was keen to trial something similar for MMIs.

### Technology selection

We initially evaluated several web-based survey tools, and while these included some excellent features we eventually ruled them out due to our previous issues with WiFi connectivity and concerns over data security. One of the authors (TJ) had developed mobile apps previously and was confident that an iPad-based solution could be developed in-house. A basic implementation plan was developed and partially supported through a learning and teaching grant from our university. Our proposed implementation process outlined staged progressions from smaller to larger scale implementations, single (MMI) to multiple (MMI, OSCE and other) assessment types and, in the case of OSCE marking, from lower to higher stakes assessments. Work on the project began in early 2015.

**Fig. 1:**
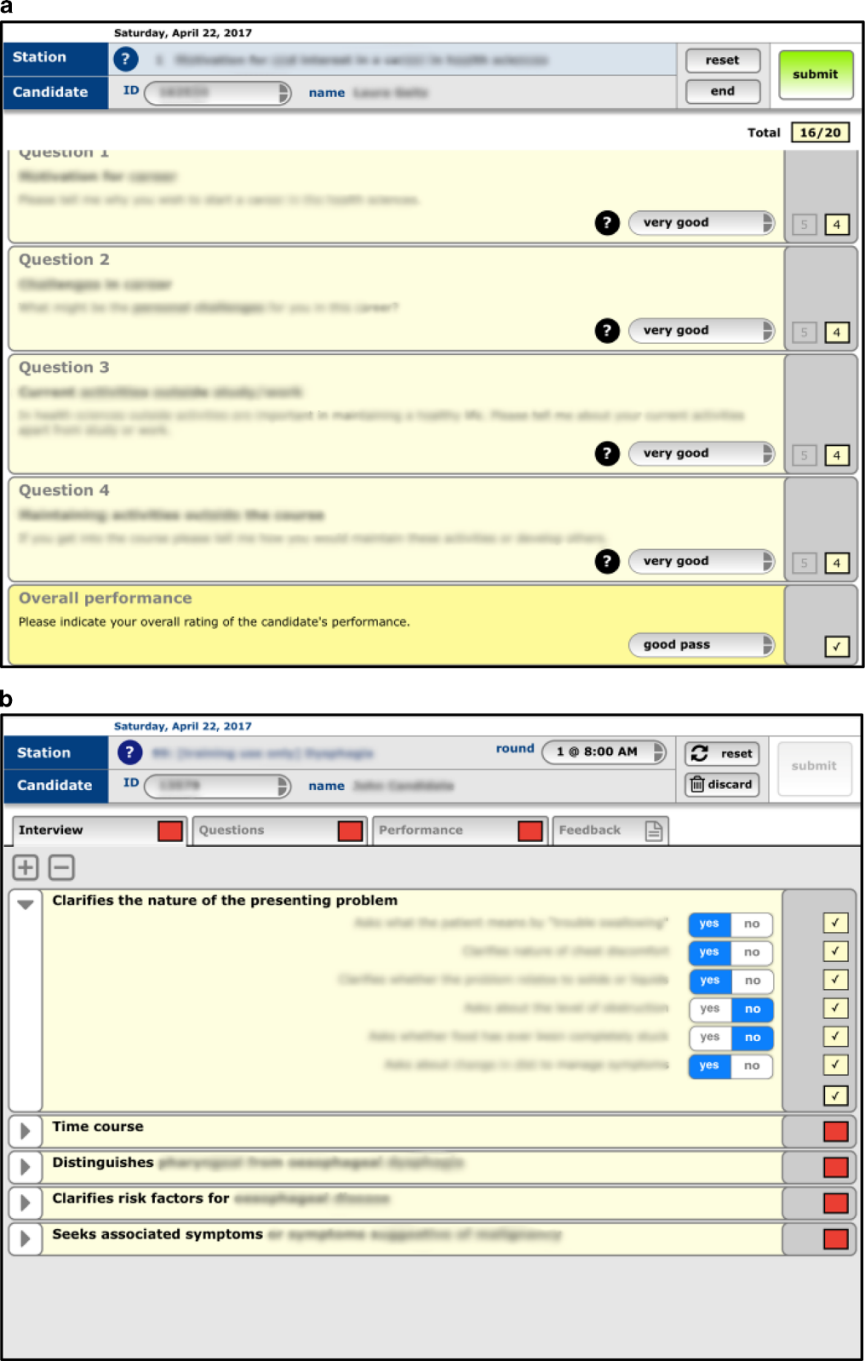
Examiner interfaces for (**a**) MMI and (**b**) OSCE marking apps

Key requirements of the MMI app were that it was: (a) effective (i. e. fit for purpose and easy to use), (b) reliable, with no loss of functionality or data (irrespective of WiFi availability), and (c) secure, with all data captured, transmitted and stored securely. To promote acceptance by experienced examiners we also felt that the app should replicate, wherever practicable, the organization of the existing paper-based information and marking forms. We adopted a form-building metaphor, similar to that used by the online survey tools we evaluated, with a view to extending its functionality (by creating additional form templates) to support OSCEs and other assessments later on. Separate configuration and administration tools were designed and developed concurrent with the iPad marking app. The configuration tool would support construction and population of forms and allocation of examiners and candidates while the administrative tool provided monitoring of submitted records and facilitated collation, processing and export of data. Rapid prototyping in conjunction with iterative internal testing and review were used to develop an initial version of the system by mid-2015 (Fig. 1a).

### Implementation

An initial small-scale ‘live’ trial was planned for August 2015 with plans for a full-scale implementation during the main MMIs several weeks later. The live trial involved a series of Skype-based MMIs for overseas or interstate candidate unable to attend the face-to-face interviews. We implemented multiple levels of data redundancy, including a mandatory but highly simplified paper record, and full paper-based marking facilities were retained as a fallback in case of major technical failure.

The live trial involved approximately 50 candidates and 10 examiners and was completed without incident. Preparations for the main MMIs proceeded and included minor refinements to the marking app and the production of user guides and training materials for examiners. As per the live trial, no major issues were experienced during the full-scale implementation. No data were lost, and all records were collated and processed within one working day of the completion of the interviews.

### Evaluation

Informal individual debriefing of examiners after the interview sessions revealed some initial anxiety and/or unfamiliarity with the marking app but almost all examiners reported being comfortable with the marking process after a couple of interviews. A few examiners reported minor usability issues, but most had been adequately resolved by support staff.

Formal evaluation of interviewers’ experiences with the app was conducted the following week via online survey. Participation was voluntary and anonymous. Of the 61 valid responses (41% effective response rate), just under half (*n* = 29) of the respondents were new to MMI assessments. Almost all (*n* = 58) reported previous experience with tablets or smartphones. Acceptance of the app with high, with around four in five respondents agreeing that the marking templates were easy to use and that the marking process was time efficient. Almost 70% of experienced examiners agreed that marking on the iPads was easier than on paper. Only 10% said it was more difficult. The app received a minor update and was used again during the 2016 MMIs.

## Stage 3: The solution part 2 (OSCE marking revisited)

Based on this success, the OSCE assessment team agreed to a demonstration/trial of a prototype OSCE marking app, with any further work being contingent on the success of this trial. Development of the prototype commenced in early 2016. Its demonstration, to around 10 participants in April 2016, involved a combination of app training, a marking exercise, and a follow-up discussion with participants. Several modifications were made based on the participants’ feedback (Fig. 1b), and after a follow-up review the assessment team agreed to proceed with a live trial during our mid-year formative OSCEs (first year students, two stations only). As with the MMIs the previous year, success during this trial would inform a more substantial implementation, during our end-of-year OSCEs. Those OSCEs are summative, multi-station, and involve all first to third year students. The delay between the two implementations would again allow time to address any major issues exposed during the live trial.

The OSCE trial proceeded without major incident. Individual examiners were again debriefed and asked to provide feedback on their experiences. A few minor technical and logistical issues were identified and after incorporating user feedback the system and implementation processes were slightly modified. The system was subsequently successfully used to mark 4 first-year and 6 third-year stations during our end-of-year OSCEs.

The next stages of our implementation process will (during 2017/18) extend the use of the OSCE marking system during the end-of-year assessments to include additional locations and students. While the system is relatively mature, these wider implementations will involve additional logistical challenges, including off-site training of staff and the movement and maintenance equipment between locations. Improvements to the system allowing us to deliver personalized post-assessment feedback for students are being trialled during 2017.

## Reflections, suggestions and lessons learned

Our recent implementations of MMI and OSCE marking systems have been successful and generally well accepted, and any resistance to electronic marking due to the initial setback appears to have dissipated. However, our adoption process has not been without challenges, and a couple of these are worth reflecting on.

The first relates to technology selection. Our systems were developed in-house. The obvious advantage here was the ability to design and develop a system that exactly matched and was responsive to our needs. However, software development can be difficult, time-consuming and expensive, and unless you have considerable experience with this process, adopting or adapting an off-the shelf solution is typically easier. The trade-off is that you may end up with a system that does not meet all your requirements. We suggest approaching other departments in your university or region to see what has worked for them, including how they dealt with any adoption problems or shortcomings.

The second is that your selected solution must be reliable. The example of unreliable WiFi connectivity, leading to the failure of our department’s initial trial of OSCE marking, is a case in point. If reliability under operational conditions is uncertain and if failure of the system could compromise data security, then an alternative solution (or an effective workaround) must be sought. Thorough testing is vital, even when implementing off-the-shelf solutions, and additional testing should be considered whenever conditions change. Never assume that success in one environment will automatically translate to others.

### Assembling an adoption team

Most adoption processes involve multiple stakeholders – in the case of electronic marking of MMIs and OSCEs this could include academics (assessment, development and oversight), examiners (end users) and administrative and technical staff (co-ordination and support). Previous authors have suggested nominating a ‘champion’ to drive this process [2]. We recommend extending and expanding this concept to recognize four interdependent adoption roles – those of innovation *advocate, champion, expert* and *sponsor* – with each of the four authors fulfilling at least one of these roles in our adoption process. The advocate supports the technology selection process and is responsible for identifying the appropriate technology, detailing the purpose it fulfils and conceptualizing how and where it might be implemented. The advocate also assumes responsibility for recruiting the champion, who facilitates and promotes the implementation. The expert designs and oversees the implementation of the innovation, typically in conjunction with the champion. The final role, of sponsor, might be unnecessary for minor innovations, but is critical for high-stakes or large-scale innovations that require overt high-level support.

Once you have your adoption team, plan your implementation process carefully. For higher-stakes innovations this will likely include measured staging and contingency planning. In our case this involved starting relatively simply (the marking of MMIs is much less complex than OSCEs) and smaller scale, and having multiple levels of data redundancy should the technology fail at any stage. Subsequent stages progress through larger more complex implementations or adaptions to different contexts, with each successive stage being contingent on previous success. Failure or major issues at any stage should be addressed immediately and successfully re-implemented before proceeding. Appropriate staging will also allow sufficient time to address any minor issues exposed during the previous stage. Our full implementation cycle will likely span four years, starting with initial development of the MMI marking system in early 2015 and ending with course-wide implementation of the OSCE marking system by late 2018. However, most technologies, particularly those that can leverage established adoption processes, can be adopted within much shorter timeframes.
